# EFFECTIVENESS OF A WEB-BASED INTERVENTION FOR LEWY BODY DEMENTIA CAREGIVERS: IMPACT BY DEPRESSION STATUS

**DOI:** 10.1093/geroni/igae098.3958

**Published:** 2024-12-31

**Authors:** Yan Su, Annie T Chen, Oleg Zaslavsky

**Affiliations:** University of Massachusetts Darmouth, Dartmouth, Massachusetts, United States; University of Washington, Seattle, Washington, United States; University of Washington, Seattle, Washington, United States

## Abstract

Lewy Body Dementia (LBD) affects 1.4 million Americans, with 80% of care provided by family members who often face significant mental health challenges. Few digital interventions are tailored specifically for LBD or offer a fully remote, asynchronous format. This pilot study evaluated the effectiveness of an 8-week web-based intervention among 39 LBD caregivers, comparing its impact on those with and without depression. The intervention included online discussions on caregiving and problem-solving skills, moderated by a trained facilitator, with participants required to post at least twice weekly. Measurements were taken at baseline, post-intervention, and one month later. Of the 39 caregivers, 21 were classified as having depression, and 18 as without depression. Among those with depression, the intervention significantly reduced depressive scores from baseline to post-intervention (𝑑 = −1.35, 95% CI: −3.36, −0.61) and to follow-up (𝑑 = −2.01, 95% CI: −3.36, −1.25). Reductions in burden (𝑑 = −0.47, 95% CI: −1.04, −0.02) and perceived stress (𝑑 = −1.12, 95% CI: −2.05, −0.54) were also observed, with sustained stress reduction at follow-up (𝑑 = −1.33, 95% CI: −3.17, −0.36). Improvements in self-efficacy (𝑑 = 0.48, 95% CI: 0.00, 1.32) and social support (𝑑 = 0.71, 95% CI: 0.08, 1.38) were noted from baseline to follow-up. In contrast, caregivers without depression showed no significant improvements in these areas, except for a modest increase in social support at follow-up (𝑑 = 0.33, 95% CI: 0.10, 0.57). This study highlights the potential of the intervention to effectively support LBD caregivers, particularly those with depression.

